# Identification of novel genetic loci GAL3ST4 and CHGB involved in susceptibility to leprosy

**DOI:** 10.1038/s41598-017-16422-1

**Published:** 2017-11-27

**Authors:** Youhua Yuan, Yuangang You, Yan Wen, Jian Liu, Huanying Li, Yumeng Zhang, Nan Wu, Shuang Liu, Shanshan Zhang, Jiazhen Chen, Jingwen Ai, Wenhong Zhang, Ying Zhang

**Affiliations:** 1 Key Laboratory of Medical Virology, Department of Infectious Diseases, Huashan Hospital, Shanghai Medical College, Fudan University, Shanghai, China; 2 Department of Clinical laboratory, Henan Provincial People’s Hospital, Zhengzhou, China; 3 Beijing Tropical Medicine Research Institute, Beijing Friendship Hospital, Capital Medical University, Beijing, China; 4 Department of Molecular Microbiology and Immunology, Bloomberg School of Public Health, Johns Hopkins University, Baltimore, Maryland USA

## Abstract

Leprosy has long been thought to have a strong genetic component, and so far, only positional cloning and genomewide association studies have been used to study the genetic susceptibility to leprosy,while whole exome sequencing (WES) approach has not yet been applied. In this study, we used WES approach on four leprosy patients and four healthy control relatives from two leprosy families. We found three new susceptible loci of leprosy, one in GAL3ST4 and two in CHGB. We went on to validate the findings of WES using 151 leprosy cases and 226 healthy controls by Sanger sequencing. Stratified by gender, GAL3ST4 was found to be the susceptible gene only for the female population, and CHGB48 and CHGB23 were susceptibile to leprosy for the male population, respectively). Moreover, the gene expression levels of the three susceptible loci were measured by real-time PCR after the stimulation by *M. leprae* antigens in the PBMC (peripheral blood mononuclear cells) of 69 healthy people. The results showed that the female subjects with high frequent genotype in GAL3ST4 had a fivefold elevated expression. We suggest the polymorphisms in GAL3ST4 in different population are associated with increased risk of leprosy.

## Introduction

Leprosy is a chronic infectious disease caused by *Mycobacterium leprae* and about 200,000 cases were reported each year^1^. The clinical features of leprosy differ greatly among individuals, and previous studies showed that the widely different clinical manifestations of leprosy contrast with the low variability of the bacillus^2^. This suggests that the host genetic variation may have played a more important role in the pathogenesis of the disease^3,4^. One of the widely-used methods to explore the differences in human genetic component is the single-nucleotide polymorphism (SNP) variation study, and previous studies have identified certain associations between SNP variations and the host susceptibility to leprosy^5,6^. Recent genome-wide association studies (GWAS) on leprosy patients have identified some susceptibility loci (*CCDC1*2*2, LACC1 (C13orf31), NOD2, TNFSF15, RIPK2, HLA-DR, HLA-DQ IL-23R)* and *RAB32* locus in the Chinese population^7–9^, which indicated the importance of host susceptibility in protection against *M. leprae*. Although the clinical progression of the disease may be associated with certain genes such as *PARK2-PACRG* ^10^, few of these associations have been confirmed in different populations^11^.

Whole exome sequencing is a new powerful strategy to discover causative genes in rare Mendelian disorders^12^. Recently, this technology combined with a filtering methodology was demonstrated as an approach to identify susceptible genes among many genetic diseases^13^. Moreover, many genetic variants of common diseases such as diabetes, hypertension and tumor were found by this approach^14–16^. However, the application of this method in the infectious diseases is scarce. In this study, we enrolled both leprosy patients and healthy controls within leprosy families to further study hostgenetic variations and their associations with the disease using whole exome sequencing.

## Results

### Results of whole exome sequencing and validation

Whole exome sequencing was carried out in four patients and four unaffected individuals from two families with leprosy. The data of patients and controls were compared utilizing database of Hapmap 1000 project and European 6500, and twenty variants were found, and fifteen variants were further validated to be correct by Sanger sequencing (Table 1).

**Table 1:** Twenty variants identified by exome sequencing.

SNP	Chromosome	Position	Gene	Major/minor Allele	Nucletide change	Protein change	Results of validation
**rs11579366**	1	145562293	ANKRD35	G/C	exon10: G1981C	E661Q	correct
**rs2852464**	12	52710721	KRT83	G/C	exon5: C837G	I279M	correct
**rs17035120**	12	104408794	GLT8D2	C/T	exon4: G109A	A37T	correct
**rs230898**	17	15217437	TEKT3	C/G	exon6: G845C	G282A	correct
**rs910122**	20	5903323	CHGB	G/A	exon4: G533A	R178Q	correct
**rs236152**	20	5903848	CHGB	C/G	exon4: C1058G	A353G	correct
**rs34813**	5	102433409	GIN1	G/A	exon5: C716T	T239M	correct
**rs3823646**	7	99757612	GAL3ST4	G/A	exon4: C1400T	A467V	correct
**rs2302445**	7	156761818	NOM1	G/A	exon10: G2336A	R779H	correct
**rs2302443**	7	156762224	NOM1	G/C	exon11: G2410C	V804L	correct
**rs12919**	7	156762248	NOM1	G/A	exon11: G2434A	V812M	correct
**rs1105929**	16	22144318	VWA3A	C/T	exon20: C1970T	T657I	correct
**rs2853533**	18	658064	C18orf56	G/C	exon1: C184G	R62G	correct
**rs17014118**	4	89319296	HERC6	T/C	exon8: T1027C	F343L	correct
**—**	11	6662745	DCHS1	−/CAG	exon2: 100insCTG	G34LG	correct
**rs79690623**	1	148004781	NBPF14	T/C	exon22: A2533G	M845V	wrong
**rs1049254**	16	88709828	CYBA	A/G	exon6: T521C	V174A	wrong
**rs200426415**	17	34587756	TBC1D3C	T/C	exon6: A328G	M110V	wrong
**rs201473096**	17	34587758	TBC1D3C	G/A	exon6: C326T	P109L	wrong
**rs200698765**	1	208272313	PLXNA2	A/C	exon6: T1069G	C537G	wrong

### Comparison of validated variants between the remaining patients from two leprosy families and healthy controls from 1000 project database

We found that five gene variants including gene GAL3ST4, CHGB48, CHGB23, GLT8D2 and ANKRD35 were more frequently reported than the other ten variants among the remaining six patients from two leprosy families by means of gene sequencing (data not shown). We found no significant difference of the frequency of five variants between patients and healthy relatives (data not shown). However, three SNP loci (GAL3ST4, CHGB48 and CHGB23) were found to have significant difference in frequency between the leprosy patients and healthy controls from database of 1000 Hapmap project (Table 2).

**Table 2:** Comparison of validated variants between remaining patients from two leprosy families and healthy controls from 1000 project database.

Gene	SNP	Minor/Major Allele	Minor Allele Frequency from 1000 project	Patient (N = 6) Minor Allele Frequency	*x* ^*2*^	*P* value
**CHGB23**	rs910122	A/G	0.45	0.75	4.242	0.039
**CHGB48**	rs236152	C/G	0.45	0.25	1.881	0.170
**GAL3ST4**	rs3823646	G/A	0.39	0.34	4.732	0.03
**GLT8D2**	rs17035120	T/C	0.34	0.5	0.723	0.395
**ANKRD35**	rs11579366	C/G	0.25	0.5	2.621	0.105
**GIN1**	rs34813	A/G	0.42	0.42	0	1
**KRT83**	rs11579366	C/G	0.25	0.17	0.102	0.749
**NOM118**	rs2302445	A/G	0.26	0.25	0	1
**C18orf56**	rs2853533	C/G	0.55	0.83	3.784	0.052
**VWA3A**	rs1105929	C/T	0.30	0.25	0.003	0.95
**HERC6**	rs17014118	T/C	0.28	0.5	1.762	0.184
**TEKT3**	rs230898	G/C	0.49	0.25	2.699	0.1
**NOM124**	rs2302443	C/G	0.26	0.25	0	1
**NOM148**	rs12919	A/G	0.26	0.25	0	1
**DCHS1**	—	−/CAG	—	0.5	—	—

### GAL3ST4 polymorphisms in cases and controls

We further expanded the testing of the GAL3ST4 (rs3823646) variants in 151 cases and 226 endemic healthy people to verify whether the difference existed. Although no difference for GAL3ST4 (rs3823646) between the patients and controls was found (Table 3), the GAL3ST4 gene polymorphism was significantly different between leprosy patients and healthy controls in female population. The frequency of AG and GG genotype for female leprosy (50% and 25%, respectively) was higher than that of female controls (39.4% and 13.2%, respectively) (OR = 3.16, *P* = 0.018; OR = 3.75, *P* = 0.027, respectively). Furthermore, the percentage of G locus for female leprosy (50%) was higher than that of female controls (32.9%)(OR = 1.68, *P* = 0.004) (Table 3). These results indicated GAL3ST4 might be the susceptible gene of female leprosy population. The Hardy-Weinberg equilibrium test for all leprosy and healthy control conformed to genetic principle (p > 0.05), indicating appropriate representation of sample collection.

**Table 3:** Genotype and allele frequency of GAL3ST4 polymorphism in leprosy cases and healthy controls stratified by gender and adjusted by ethnicity and age.

Gender	Group	Genotype					HWE test p
		AA(%)	GG(%)	AG(%)	A(%)	G(%)	
**Male**	Leprosy (N = 99)	49(49.5)	9(9.1)	41(41.4)	139(70.2)	59(29.8)	0.92
	Control (N = 112)	48(42.9)	15(13.4)	49(43.7)	145(64.7)	79(35.3)	0.66
	OR (95%CI)	1.01 (0.51–2.0)	1.46 (0.51–4.2)	0.98 (0.5–1.9)	1.1 (0.69–1.8)	0.89 (0.6–1.4)	
	*P*	0.5	0.97	0.48	0.64		
**Female**	**Group**	**AA(%)**	**GG(%)**	**AG(%)**	**A(%)**	**G(%)**	
	Leprosy (N = 52)	13(25)	13(25)	26(50)	52(50)	52(50)	1
	Control (N = 114)	54(47.4)	15(13.2)	45(39.4)	153(67.1)	75(32.9)	0.26
	OR (95%CI)	0.3 (0.12–0.74)	3.75 (1.2–12.1)	3.16 (1.2–8.2)	0.58 (0.28–0.85)	1.68 (1.2–3.1)	
	*P*	0.009	0.027	0.018	0.004		
**Total**	**Group**	**AA(%)**	**GG(%)**	**AG(%)**	**A(%)**	**G(%)**	
	Leprosy (N = 151)	62(41.1)	22(14.6)	67(44.3)	191(63.2)	111(36.8)	0.58
	Control (N = 226)	102(45.1)	30(13.3)	94(41.6)	298(65.9)	154(34.1)	0.26
	OR (95%CI)	0.95 (0.46–1.9)	0.72 (0.43–1.2)	0.68 (0.33–1.4)	0.78 (0.51–1.1)	1.26 (0.89–1.8)	
	*P*	0.88	0.219	0.31	0.194		

### Gene expression of GAL3ST4 in different genotypes of female population

**Figure 1:**
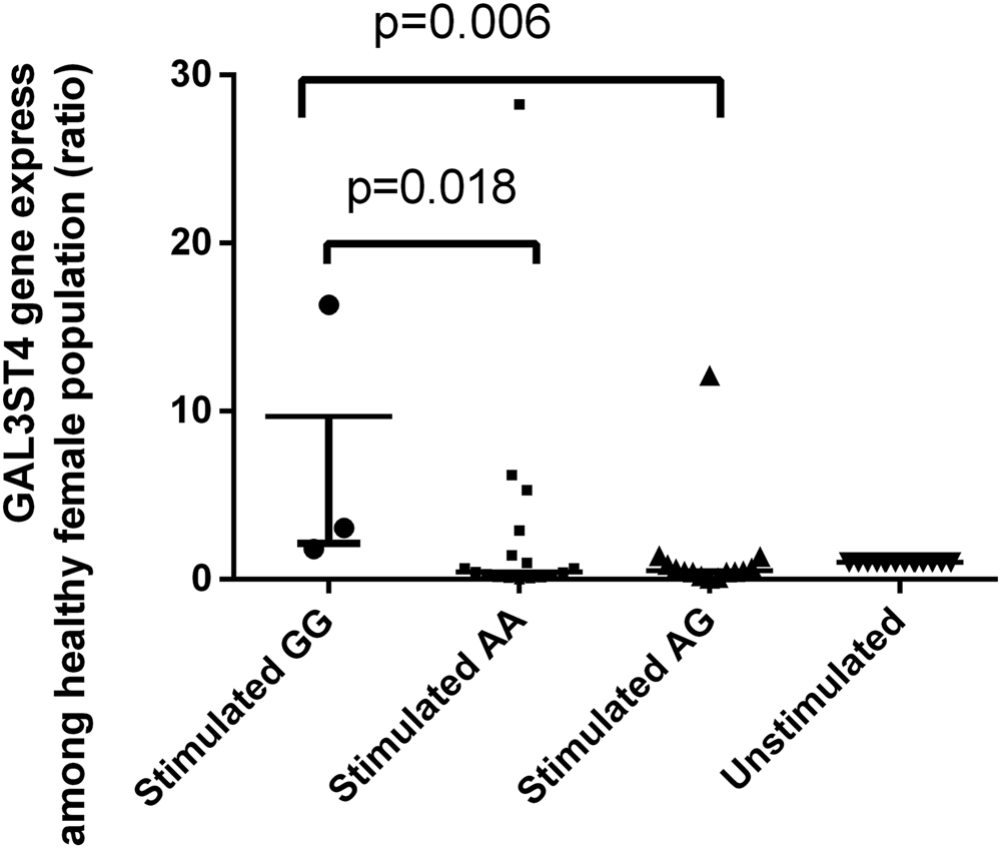
Plot of GAL3ST4 expression in response to stimulation with *M. leprae* antigens. Data were derived from PBMCs from 35 female healthy people stimulated with *M.leprae* antigens. GAL3ST4 transcript levels are revealed as ratios and shown in median with interquantile range; p values are calculated using the Manny-Whitney U test.

To assess whether the three kinds of genotype of GAL3ST4 on the susceptibility of leprosy in female patients is exerted through changes in GAL3ST4 expression, we performed an *in vitro M. leprae* antigen stimulation assay. PBMCs from 28 female healthy subjects (12 AA, 4 GG, and 12 AG) were stimulated with and without *M. leprae* antigens for 12 hours, respectively, and the expression level of the GAL3ST4 mRNA was quantified by real-time PCR. As shown in Fig. 1, significantly higher GAL3ST4 expression was observed following *in vitro* antigen stimulation in GG homozygotes compared to AA homozygotes (p = 0.018) or AG heterozygotes (p = 0.006), respectively. (Fig. 1). These results revealed the female population with G allele were more readily infected with *M. leprae* by means of mediating expression of GAL3ST4 in monocytes/macrophages. Therefore, we postulate a positively selected polymorphism in the GAL3ST4 exon region of genome for female population might be associated with the susceptibility to *M. leprae* infection by upregulating GAL3ST4 expression.

### CHGB48 polymorphisms in cases and controls

We further validated whether the difference exists for the CHGB48 SNP(rs236132) in previously collected 140 leprosy patients and 190 endemic healthy people. There was no significant difference for CHGB48 polymorphisms between the patients and the controls (Table 4). However, CHGB48 polymorphism in male population was found to be significant between leprosy patients and healthy controls. The distribution of GC genotype for male leprosy (50%) was higher than that of male control (39.4%)(OR = 2.85, *P* = 0.011). Meanwhile, the frequency of CC locus in male leprosy (16.3%) was lower than that of male controls (22.3%)(OR = 0.38, *P* = 0.03). These results indicated G allele of CHGB48 might be the susceptible site of leprosy for male population. The Hardy-Weinberg equilibrium test for all leprosy patients and healthy controls indicated the samples were representative of population.

**Table 4:** Genotype and allele count of CHGB48 polymorphism in leprosy cases and healthy controls stratified by gender and adjusted by ethnicity and age.

Gender	Group	Genotype					HWE Test P
		CC %	GC %	GG %	G %	C %	
**Male**	Leprosy (N = 92)	15 (16.3)	46 (50)	31 (33.7)	108 (58.7)	76 (41.3)	0.76
	Control (N = 94)	21 (22.3)	37 (39.4)	36 (38.3)	109 (58.0)	79 (42.0)	0.06
	O R (95%CI)	0.88 (0.3–2.5)	2.85 (1.0–7.9)	0.38 (0.16–0.9)	0.99 (0.52–1.6)	1.1 (0.67–1.8)	
	*P*	0.81	0.043	0.03	0.69		
**Female**		**CC %**	**GC %**	**GG %**	**G %**	**C %**	
	Leprosy (N = 48)	10 (20.8)	29 (60.4)	9 (18.8)	47 (48.9)	49 (51.1)	0.14
	Control (N = 96)	22 (22.9)	56 (58.3)	18 (18.8)	92 (47.9)	100 (52.1)	0.09
	O R (95%CI)	0.7 (0.3–4.9)	1.1 (0.3–3.1)	0.77 (0.17–3.6)	1.2 (0.65–2.4)	0.832 (0.43–1.6)	
	*P*	0.623	0.916	0.739	0.579		
**Total**		**CC %**	**GC %**	**GG %**	**G %**	**C %**	
	Leprosy (N = 140)	25 (17.9)	75 (53.5)	40 (28.6)	155 (55.4)	125 (44.6)	0.32
	Control (N = 190)	43 (22.6)	93 (48.9)	54 (28.5)	201 (52.9)	179 (47.1)	0.86
	O R (95%CI)	0.57 (0.3–1.1)	1.67 (0.8–3.3)	0.99 (0.48–2.3)	1.05 (0.75–1.5)	0.99 (0.7–1.4)	
	*P*	0.085	0.169	0.888	0.784		

### Gene expression of CHGB48 in different genotypes of male population

**Figure 2:**
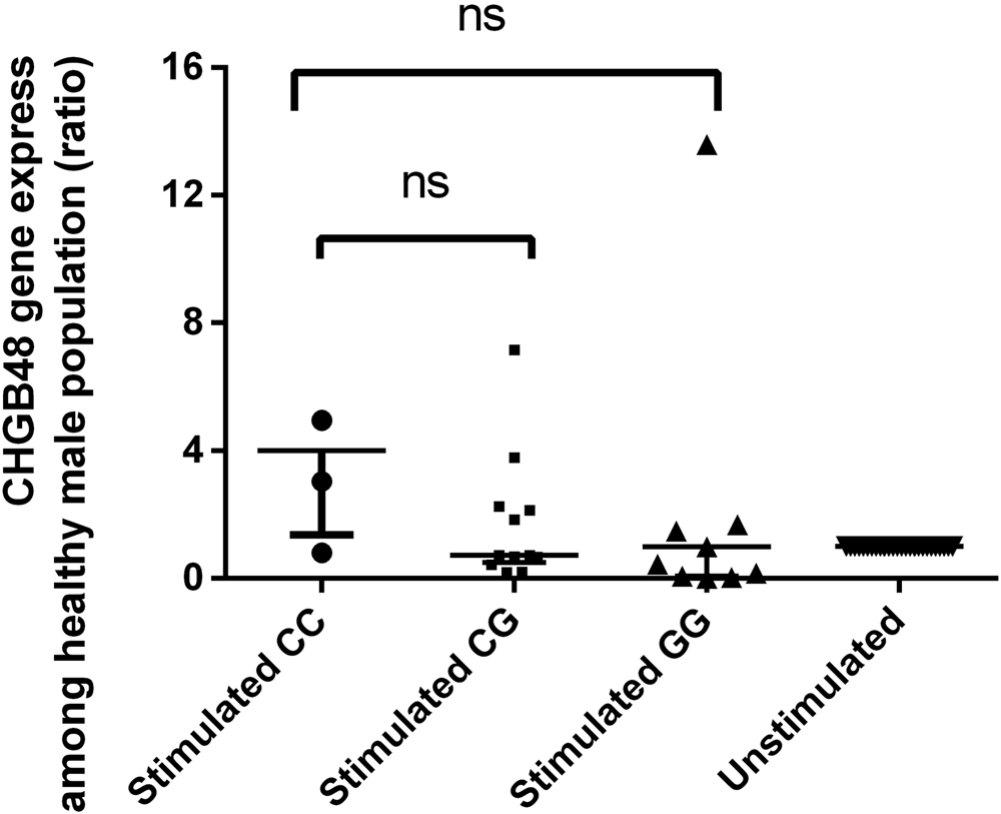
Plot of CHGB48 expression in response to stimulation with *M*. *leprae*antigens. Data were derived from PBMCs from 28 male healthy people stimulated with *M*. *leprae* antigens. CHGB48 transcript levels are revealed as ratios and shown in median with interquantile range; *p* values are calculated using the Manny-Whitney U test. ns: no significance.

To explore whether the three kinds of genotype of CHGB48 on the susceptibility of leprosy in male patients is exerted through changes in CHGB48 expression, we conducted an *in vitro M. leprae* antigen stimulation assay. Specifically, PBMCs from 28 male healthy subjects (12 GG, 5 CC, and 11 CG) were stimulated with and without antigen of *M. leprae* for 12 hours, respectively, and the expression level of the CHGB48 mRNA was quantified by real-time PCR. As shown in Fig. 2, no difference for CHGB48 expression was observed following *in vitro* antigen stimulation among different genotypes (Fig. 2). These results found no association between the susceptibility of CHGB48 locus and gene expression among the male population.

### CHGB23 polymorphisms in cases and controls

The 145 leprosy patients and 189 endemic healthy people were collected to validate whether the difference exist for the CHGB23 SNP(rs910122). The results showed not only significant difference for CHGB23 between the patients and controls (Table 5), but also, CHGB23 gene polymorphism in male population was found to be different between leprosy patients and healthy controls. The percent of AG genotype for leprosy (51.7%) was higher than that of control (46.6%) (OR = 1.69, *P* = 0.058). Meanwhile, the prevalence of AA genotype for male leprosy (29.7%) was lower than that of control (33.9%)(OR = 0.214, *p* = 0.029). Furthermore, stratified by gender, the frequency of AA for male leprosy (34.4%) was lower than that of male control (44.7%) (OR = 0.142, *P* = 0.003). Accordingly, the percentage of allele G for male leprosy (42.7%) was much higher that of A for male controls (35.1%)(OR = 11.73, *p* = 0.001). These results revealed G allele of CHGB23 might be the susceptible site of leprosy infection in the male population. The cases and healthy controls conformed to the Hardy–Weinberg equilibrium test, (p > 0.05), indicating the sample selection has appropriate representation.

**Table 5:** Genotype and allele count of CHGB23 polymorphism in leprosy cases and healthy controls stratified by gender and adjusted by ethnicity and age.

Gender	Group	Genotype					HWE test P
		AG %	GG %	AA %	A %	G %	
**Male**	Leprosy (N = 96)	44 (45.8)	19 (19.8)	33 (34.4)	110 (57.3)	82 (42.7)	0.53
	Control (N = 94)	38 (40.4)	14 (14.9)	42 (44.7)	122 (64.9)	66 (35.1)	0.27
	O R (95%CI)	1.6 (0.8–3.3)	1.72 (0.7–4.5)	0.142 (0.22–0.93)	0.087 (0.32–0.9)	11.73 (1.1–2.9)	
	*P*	0.213	0.239	0.003	0.001		
**Female**		**AG %**	**GG %**	**AA %**	**A %**	**G %**	
	Leprosy (N = 49)	31 (63.3)	8 (16.3)	10 (20.4)	51 (52)	47 (48)	0.06
	Control (N = 95)	50 (52.6)	23 (24.2)	22 (23.2)	94 (49.5)	96 (50.5)	0.61
	O R (95%CI)	1.82 (0.24–1.8)	0.339 (0.46–4.9)	0.46 (0.22–3.2)	1.1 (0.61–1.8)	0.917 (0.514–1.63)	
	*P*	0.263	0.573	0.671	0.768		
**Total**		**AG %**	**GG %**	**AA %**	**A %**	**G %**	
	Leprosy (N = 145)	75 (51.7)	27 (18.6)	43 (29.7)	161 (55.5)	129 (44.5)	0.57
	Control (N = 189)	88 (46.6)	37 (19.6)	64 (33.9)	216 (57.1)	162 (42.9)	0.50
	O R (95%CI)	1.69 (0.35–1.0)	0.79 (0.38–1.6)	0.214 (0.22–0.85)	0.747 (0.49–1.1)	1.4 (0.99–2.10)	
	*P*	0.058	0.53	0.027	0.166		

### Gene expression of CHGB23 in different genotypes of male population

**Figure 3:**
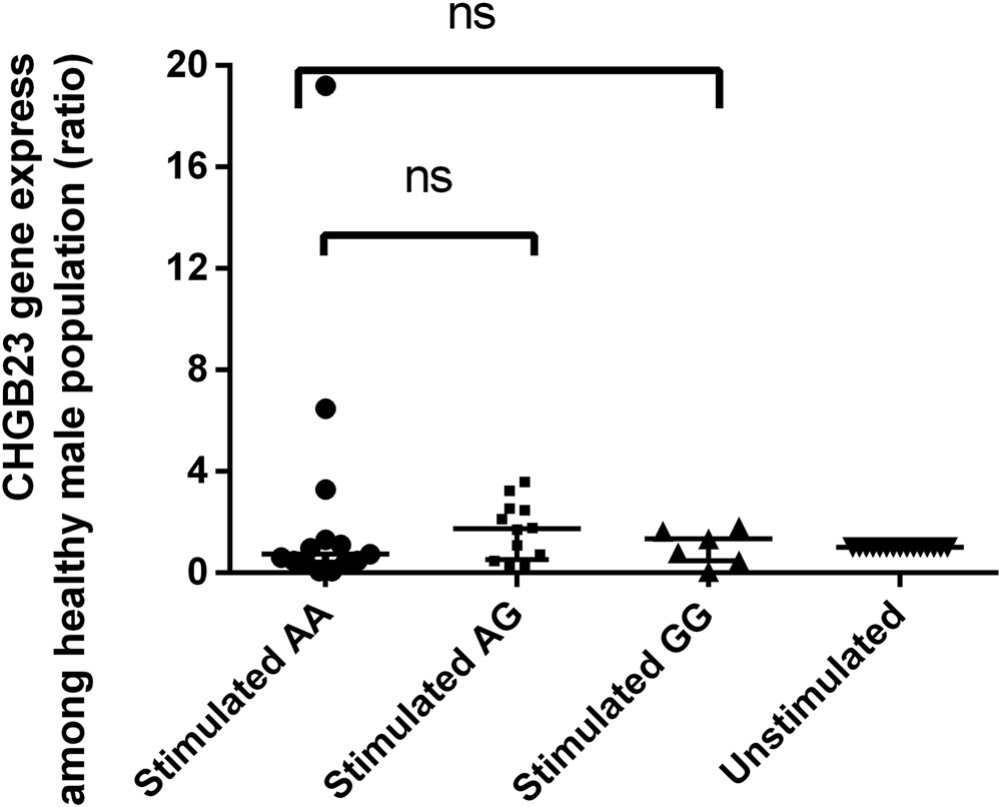
Plot of CHGB23 expression in response to stimulation with *M.leprae* antigens. Data were derived from PBMCs of 34 male healthy people stimulated with *M.leprae* antigens. CHGB23 transcript levels are revealed as ratio and shown in median with interquantile range; p values are calculated using the Manny-Whitney U test. ns: no significance.

To examine whether the three kinds of genotype of CHGB23 on male leprosy susceptibility is exerted through changes in CHGB23 expression, we conducted an *in vitro M. leprae* antigen stimulation assay. Specifically, PBMCs from 34 male healthy subjects (7 GG, 15AA, and 14AG) were stimulated with or without *M. leprae* antigens for 12 hours, respectively. The ratio of the RNA expression amount with or without antigen stimulation was regarded as the gene expression level, and CHGB23 RNA expression abundance was quantified by real-time PCR. As shown in Fig. 3, no significant difference for CHGB23 expression was observed following *in vitro* antigen stimulation among different genotypes (Fig. 3). These results revealed no relationship for the male population with G allele in CHGB23 gene between susceptibility and gene expression of leprosy.

### C13orf31 polymorphisms in cases and controls

In order to verify the reliability of three leprosy susceptible loci (GAL3ST4,CHGB48 and CHGB23) found in our experiment, the expression of the known leprosy susceptibility gene C13orf31 discovered in Chinese population^9^, was studied in the same group of leprosy patients and endemic healthy controls. As shown in Table 6, there was a significant difference for genotype between female multibacillary and paucibacillary. The frequency of genotype AG for female multibacillary patients (58.3%) was significantly higher than that of paucibacillary patients (8.3%) (OR = 16, p = 0.03). Additionally, the frequency of G allele for female multibacillary patients (50.2%) was also higher than that of female paucibacillary patients (20.8%). Meanwhile, the percentage of genotype GG in all leprosy patients (13.1%) was more than that of healthy controls (7.5%) (OR = 3.3, p = 0.045) (Table 7). Accordingly, the frequency of G allele in all leprosy patients (36.3%) was higher than that of healthy controls (24.3%) (OR = 1.79, P = 0.024). These data indicated G allele of C13orf31 gene was not only the susceptibility locus for female multibacillary, but also for the leprosy patients. The result was consistent with the report discovered by genomewide association study.

**Table 6:** Genotype and allele count of C13orf31 polymorphism in multibacilary and paucibacilary patients stratified by gender and adjusted by ethnicity and age.

Gender	Clinical type	Genotype				
		AA (%)	GG (%)	AG %	A (%)	G (%)
**Male**	Multibacillary (N = 38)	14 (36.8)	4 (10.5)	20 (52.6)	48 (63.2)	28 (36.8)
	Paucibacillary (N = 22)	9 (40.9)	2 (9.1)	11 (50)	29 (65.9)	15 (34.1)
	OR (95%CI)	0.34 (0.38–3.6)	0.68 (0.1–4.8)	0.91 (0.28–2.95)	0.32 (0.5–2.6)	1.75 (0.4–2)
	*P*	0.362	0.7	0.88	0.202	
**Female**	Multibacillary (N = 12)	2 (16.7)	3 (25)	7 (58.3)	11 (45.8)	13 (54.2)
	Paucibacillary (N = 12)	9 (75)	2 (16.7)	1 (8.3)	19 (79.2)	5 (20.8)
	OR (95%CI)	0.034 (0.001–0.9)	3.5 (0.08–14)	16 (1.47–167)	0.223 (0.06–0.8)	4.5 (1.3–16)
	*P*	0.042	0.57	0.03	0.017	
**Total**	Multibacillary (N = 50)	16 (32)	7 (14)	27 (54)	59 (59)	41 (41)
	Paucibacillary (N = 34)	18 (52.9)	4 (11.8)	12 (35.3)	48 (70.6)	20 (29.4)
	OR (95%CI)	0.93 (0.78–5.4)	0.894 (0.38–6.6)	0.97 (0.63–4.5)	0.95 (0.81–3.3)	1.1 (0.3–1.2)
	*P*	0.935	0.914	0.97	0.933

**Table 7:** Genotype and allele count of C13orf31 polymorphism in leprosy and healthy people stratified by gender and adjusted by ethnicity and age.

Gender	Group	Genotype					HWE Test P
		AA (%)	GG (%)	AG (%)	A (%)	G (%)	
**Male**	Leprosy (N = 60)	23 (38.3)	6 (10)	31 (51.7)	77 (64.2)	43 (35.8)	0.34
	Control (N = 31)	18 (58)	2 (6.5)	11 (35.5)	47 (75.8)	15 (24.2)	0.86
	OR (95%CI)	0.62 (0.8–5.4)	2.5 (0.7–5.4)	2.1 (0.1–4.9)	0.9 (0.88–3.8)	1.12 (0.28–1.2)	
	*P*	0.447	0.317	0.163	0.77		
**Female**	Leprosy (N = 24)	11 (45.8)	5 (20.9)	8 (33.3)	30 (62.5)	18 (37.5)	0.16
	Control (N = 49)	29 (59.2)	4 (8.2)	16 (32.7)	74 (75.5)	24 (24.5)	0.41
	OR (95%CI)	1.5 (0.56–4.4)	2.5 (0.07–1.3)	2.14 (0.27–2.6)	2.3 (0.2–1.2)	1.3 (0.8–4)	
	*P*	0.429	0.06	0.217	0.54		
**Total**	Leprosy (N = 84)	34 (40.5)	11 (13.1)	39 (46.4)	107 (63.7)	61 (36.3)	0.45
	Control (N = 80)	47 (58.8)	6 (7.5)	27 (33.7)	121 (75.7)	39 (24.3)	0.45
	OR (95%CI)	0.6 (0.25–1.1)	3.3 (1.1–10)	1.63 (0.78–3.4)	0.56 (0.3–0.95)	1.79 (1.1–3.1)	
	*P*	0.229	0.045	0.192	0.024		

## Discussion

Up to now, there have been three methods to identify the susceptible genes of leprosy. Positional cloning is the first conducted approach, and *PARK2* and *PACRG* were the first susceptible genes of leprosy by this approach^10^. The genomewide association studies for leprosy were later conducted and found about eleven leprosy susceptibility genes^7–9^. Besides, a few susceptible genes of leprosy were discovered by comparison of different frequency between leprosy cases and controls^17,18^. Fifty nine susceptible genes of leprosy have been identified by the above three kinds of method^19–33^. However, whole exome sequencing has not been utilized to find the susceptibility genes for leprosy. In this study, we first reported three susceptible loci using whole exome sequencing. One site was located in the GAL3ST4 gene, and the other two sites were located in the CHGB gene.

Interestingly, we identified that the three loci are closely related to gender. After the stimulation by *M. leprae* antigens on PBMCs from healthy people, we observed that the female individuals with GG genotype had a significantly elevated GAL3ST4 expression levels than those with AA and AG. These results were in agreement with the effect we observed on genetic susceptibility, suggesting that the polymorphisms of these genes are associated with their expression after *M. leprae* infection.

GAL3ST4 gene is located on human chromosome 7, and its coding protein is galactose sulfonium transferase, which participates in glycoprotein synthesis, metabolism, and cell signal transduction^34^. Studies have shown that the mutations of this gene can lead to pectus excavatum^35^, and the gene may also be involved in childhood bone mature process. Our research shows that the gene may be one of the susceptibility loci in female leprosy patients. The female population who have G allele homozygous mutation in GAL3ST4 are more likely to be infected with *M. leprae*.

CHGB gene is situated on chromosome 20, encoding secreted protein tyrosine sulphation peptide, a regulation peptide precursor which is rich in the endocrine and nerve cells. This protein can function as hormone and also participate in protein binding^36^. We found the two polymorphisms of this gene are associated with leprosy. The heterozygous mutation of G5903848C in chromosome 20(CHGB48) is a susceptibility loci for leprosy in male population. However, GG homozygous mutations in this site may be a protective locus for male against leprosy, indicating that the mutation of the locus G to C is likely important. When G is mutated to C in this locus, it will lead to the change of amino acid from glycine to alanine, and may cause the change of protein function secreted by endocrine cells. The other leprosy susceptibility locus is located at a different site in the same gene CHGB23. The GG homozygous site is also one of the leprosy susceptibility loci for male population. When A is mutated to G at site 23, it will lead to the change of glutamine to arginine. The CHGB gene may play an important role for leprosy susceptibility, and men with GG homozygous are less likely to suffer from leprosy.

Due to the small number of the patients and healthy controls in our study, we selected the known leprosy susceptible gene C13orf31 as the positive control in the same group of leprosy patients and healthy people. The result confirmed that this gene is indeed a susceptibility locus for leprosy, and it is also specifically susceptible in female population. This finding revealed the reliability and accuracy of the other three susceptible loci for leprosy.

In conclusion, the GAL3ST4 and the CHGB allele variants 23 and 48 are novel genetic loci involved in susceptibility to leprosy among female and male population, respectively. However, these observations need to be further confirmed and validated in larger populations. Additionally, our genetic findings combined with the expression of GAL3ST4 and CHGB in PBMC, point strongly to an important function of secretogranin and galactose-3-O-sulfotransferase involved in the synthesis and metabolism of glycoprotein in the study of leprosy pathogenesis. Overall, our study demonstrates a significant association of GAL3ST4 and CHGB polymorphisms with leprosy and suggests that these gene polymorphisms may be a contributing factor in leprosy susceptibility. Further studies on functional characterization of SNPs may shed light on the association of these polymorphisms with leprosy.

## Materials and Methods

### Ethics statement

The study was designed and performed according to the Helsinki declaration and was approved by the Ethics Committees of the Beijing Tropical Medicine Research Institute. All patients and healthy blood donors provided written informed consent to participate in this study.

### Subjects and samples

**Figure 4:**
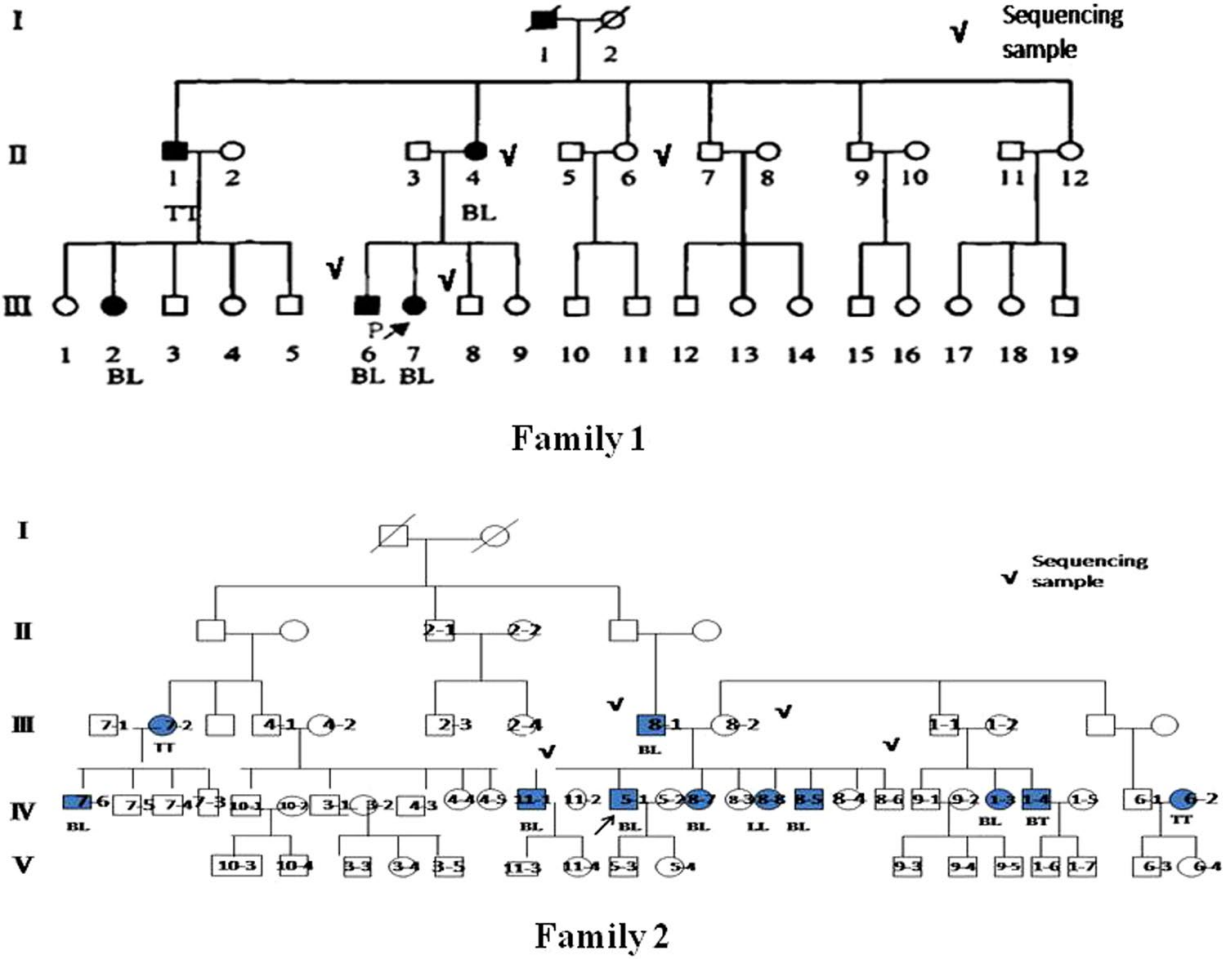
The pedigrees of the two families affected by leprosy included in the present study. Filled-in symbols indicate individuals with leprosy, empty circles indicate unaffected individuals, and symbols with a slash through them indicate deceased individuals. √, exome sequencing individuals. BL, LL, BT and TT are the clinical pathological type of leprosy. Arrows indicate the probands of the families.

The clinical data and blood samples were obtained from one Chinese Han family that included5 leprosy patients and 26 unaffected individuals from, and another Chinese Zhuang ethnic minority family that included 11 patients and 52 unaffected individuals. All individuals with leprosy met the World Health Organization diagnostic criteria for the disease. Four affected individuals (II-4, III-6,8-1 and 11-1) and four unaffected individuals (II-6, III -8,8-2 and 8-6) from family 1 and family 2 were selected for whole exome sequencing (Fig. 4), and the remaining individuals from two families and other leprosy patients outside the two families and controls from the same endemic region were recruited for Sanger sequencing. A total of 151 leprosy cases and 226 controls were recruited for this study. All patients were Han ethnic Chinese or ethnic minorities from Yunnan province, and controls were from the same endemic region. Demographic and other selected characteristics of the cases and controls were presented (Table 8). Cases and controls showed statistically significant differences with regard to age, gender and ethnicity (P < 0.05). As for the leprosy patients, 92 were multibacillary, and 59 were paucibacillary.

**Table 8:** General characteristics of the subjects.

	Leprosy cases (n = 151)(%)	Endemic healthy controls(n = 226) (%)	*P* value
**Age(Mean ± SD)**	41.6 ± 16.7	35.9 ± 15.2	<0.05
**Ethnicity**			<0.05
**Han**	56 (37.1)	108 (47.8)	
**Yi**	22 (14.6)	44 (19.5)	
**Miao**	37 (24.5)	17 (7.5)	
**Others**	36 (23.8)	57 (25.2)	
**Gender**			<0.05
**Male**	99 (65.6)	112 (49.6)	
**Female**	52 (34.4)	114 (50.4)	
**Leprosy classifications**			
**multibacillary**	92 (60.9)		
**paucibacillary**	59 (39.1)		

### Whole-exome sequencing

Genomic DNA was extracted from peripheral blood with EDTA anticoagulation using a QIAamp DNA Blood Mini kit (Qiagen). Purified DNA was analyzed on a ND-8000 spectrophotometer (Nanodrop, Technologies, Wilmington, DE), and Qubit (Invitrogen, CA, USA), to determine the quantity. DNA samples were used only if the 260/280 ratio was above 2.0 and no smear on the agarose. High quality DNA (1 μg) was used as the starting material. The DNA was fragmented by Bioruptor Sonicator (Diagenode, USA). The Truseq DNA sample preparation kit was used for end repair, dA tailing, adaptors ligation and DNA fragments enrichment. The TruSeq Enrichment kit was used to capture exome or custom sequences of a human DNA library. After two rounds hybridization and wash, the DNA exome library was subjected toHiSeq. 2500 sequencing platform to ensure that each sample was covered to a depth of at least 50×. Raw image files were processed using Ilumina Pipeline version 1.9 for base calling with default parameters, and the sequences of each individual were generated as 90-bp paired-end reads. BWA was used to align the clean reads to the UCSC human reference genome (hg19). On the basis of BWA alignment results, GATK software was used to assemble the consensus sequence and call genotypes in target regions. Insertions and deletions (indels) in the exome regions were identified using GATK software. Public databases available from dbSNP137, the 1000 Genomes Project, and the NHLBI Exome Sequencing Project (ESP 6500) were used for analysis of the results.

### Sanger sequencing

Sanger sequencing was performed to confirm the variants found by whole-exome sequencing. The PCR primers (sequences and conditions provided in Table 9) were designed to amplify the variants. PCR amplification was carried out using a thermal cycler PCR System (Takara) using standard conditions. PCR products were examined by 1% agarose gel electrophoresis, which were then sequenced directly by Shanghai Bioshine company.

**Table 9:** PCR primers and conditions designed for validation and gene expression of variants identified by exome sequencing.

SNP	Gene	Oligonucleotide primer(5′-3′)	Product size	Annealing temperature (°C)
**rs11579366**	ANKRD35	F:GGAGGAGTTAGGGGAGTTGG	281	60
		R:CTTTGCTTTGTGCACTCCTCT		
**rs2852464**	KRT83	F:TACCTCACATCCCTCCCACT	246	59
		R:TCCTTCCTTTCCCCACCT		
**rs17035120**	GLT8D2	F:GTCATGGAAGGCACCTACTCGCACT	475	62
		R:GAGGCCCCACACCATACCTACTGA		
**rs230898**	TEKT3	F:CCAGAGAGTGCTCGTTTTGACTGAGA	472	64
		R:ACCACAGAACGTGCTTGCTTACTGGA		
**rs910122**	CHGB	F:CTATCCCTCCGACAGCCA	226	60
		R:TCTGGCCACTAACTCCTCTTT		
**rs236152**	CHGB	F:GTTTAGGGGAAAAGAGGGACC	203	60
		R:TCTCTTGTCCTCCTCATCCCA		
**rs34813**	GIN1	F:ATTGAAGGCAAATGAAACAGC	328	60
		R:GCCTGGGTGACTGAGCAA		
**rs3823646**	GAL3ST4	F:TGGCTGCTCTTCCACCTAAA	220	60
		R:CCTTCCAGTTTGGGTCAGC		
**rs2302445**	NOM1	F:TTCTTAAATCGGGTAGAGTGGG	292	60
		R:AAAACAATCTCTTCATTTCTCTTCC		
**rs2302443**	NOM1	F:TGTGCCCCACCCTTTCCGACGAGA	598	62
		R:ACCAAACATTCACACGCAGGCAAC		
**rs12919**	NOM1	F:TGTGCCCCACCCTTTCCGACGAGA	598	62
		R:ACCAAACATTCACACGCAGGCAAC		
**rs1105929**	VWA3A	F:CCCACCAGCCTACACTCAGTGCCTA	549	58
		R:ATCTCAAGCGATCTGACCACGTT		
**rs2853533**	C18orf56	F:GAATGGTCCACAGGGGAAAACGG	519	64
		R:TGGGGCAGATCCAACACATCCTC		
**rs17014118**	HERC6	F:ATGCATTTGTAGTCAAAATCCATT	662	53
		R:ACATGCCACCCCTGCTACACA		
**—**	DCHS1	F:AGGCACAGCTGGACTACCCTT	425	66
		R:GCCACTCGCACTGTAACTTCTACGG		
**rs2275606**	C13orf31	F:GCGATTAGGGTCTAGCACCGACA	566	63
		R:AATAGCCTGCTTTGGGGACCTT		
**—**	GAPDH	F:CCCCTTCATTGACCTCAACTAC	103	60
		R:GATGACAAGCTTCCCGTTCTC		

### Gene expression analysis

Whole blood was collected from 48 healthy volunteers by venipuncture in Vacutainer tubes containing EDTA (BD company USA), and peripheral blood mononuclear cells (PBMCs) were separated on lymphocyte separation medium (CEDARLANE Ltd, Cat. No. CL5020). PBMCs (2 × 10^6^ cells/ ml) were cultured for 12 hours at 37 °C and 5% CO_2_ in RPMI 1640 containing antigens of *M. leprae* (10 μg/ml, Whole Cell Sonicate, NR-19329, NIH, USA) and without the antigens as a control. Cells were plated in 12-well cell culture plates and were collected to add TRIZOL (Invitrogen) and reserved at −80 °C for gene expression analysis. RNA was extracted from cultured PBMCs with or without antigen stimulation by using the acid guanidium thiocyanate–phenol–chloroform method. The RNA was treated with RNase-free water (Shanghai Biotechnology Company). RNA (500 ng) was reverse transcribed into first-strand cDNA in a 10 μl final volume containing 100 μM random hexanucleotide primers, 50 μM oligonucleotide, and 0.5 μl RT Enzyme Mix I (TAKARA). cDNA quantification for GAL3ST4, CHGB23, CHGB48 and GAPDH was performed by real-time PCR (7500 PCR system, life Applied Biosystems, Foster City, CA, USA). Reactions were performed using a SYBR Green PCR mix (TAKARA). The primers used are listed in Table 9. Results were expressed as ⊿Ct between the target gene and the GAPDH housekeeping mRNA, and presented as ratios as 2^−⊿⊿Ct^ between the genes stimulated or unstimulated with *M. leprae* antigens.

### Statistical analysis

The SPSS statistical software package ver.20.0 (SPSS Inc., Chicago, USA) was used for statistical analysis. The gene polymorphisms were tested for deviation from Hardy–Weinberg equilibrium (HWE) by comparing the observed and expected genotype frequencies using the Pearson chi-square test by HWE 2.1 software. The comparison of gene expression levels between cases and controls was performed using the Mann-Whitney U test. The chi-square test was used to compare the difference of ethnicity and gender between cases and controls. For SNP analysis, the genotype and allele frequencies of GAL3ST4 and CHGB were compared between groups using multivariate logistic regression analysis. *p* value, odds ratios (OR) and 95% confidence intervals (CIs) after adjusting age and ethnicity were calculated using binary unconditional logistic regression. *P* values less than 0.05 were considered statistically significant.
